# Expanded Utilization of the Digital Atasoy Flap

**DOI:** 10.1097/GOX.0000000000002049

**Published:** 2018-12-05

**Authors:** Enrique J. Viciana, Anne-Sophie Lessard

**Affiliations:** From the * Division of Plastic, Reconstructive and Aesthetic Surgery; † Division of Plastic, Reconstructive and Aesthetic Surgery, University of Miami Leonard M. Miller School of Medicine, Miami, Fla.

## Abstract

**Background:**

The Atasoy, or Kleinert flap, is well-known to hand surgeons. This triangular volar V-Y flap is frequently used for reconstruction of fingertip amputations with exposed bone. It is indicated in transverse amputations or in dorsal oblique amputations, providing replacement of an area of skin and subcutaneous tissues with sensibility. Originally, this flap was not recommended for use in volar oblique amputations (greater volar tissue loss). With the described modifications and recommendations, modest volar oblique amputations can be closed in a single stage, obviating a 2-stage procedure.

**Methods:**

With the described technical modifications, modest volar oblique amputations can be closed. An injury that previously may have required a 2-stage procedure can be closed in a single stage.

**Results:**

The elevation of the flap was originally described as a dissection at the volar periosteum from a distal approach. This distal dissection is no longer recommended, as it does not create advancement. Beasley indicated the need for division of the vertical fibrous septa proximally for flap mobilization. This technique description emphasizes the importance of this division of the fibrous septa rather than stretching. Careful treatment of the remaining bone is stressed. Coverage of the nail bed is not recommended.

## INTRODUCTION

The Atasoy triangular V-Y flap, described first in 1970,^1–5^ is an especially useful single stage procedure for reconstructing complex fingertip injuries, which provides “good contour and padding … and most important of all, preserves normal sensation.”^4^ Although originally not recommended for volar oblique losses, new technique modifications allow its use in modest cases of volar oblique losses. The performance of the Atasoy flap described here differs from the original description in 5 significant ways.

1. The original direction of dissection was from distal to proximal. Our recommendation is for a dissection from lateral to medial.
2. The distal dissection along the periosteum and flexor tendon sheath is no longer recommended. Our recommendation is for dissection at the mid flap level.
3. The fibrous septa that tether the flap are divided, not simply stretched, allowing for maximal advancement. This additional advancement can cover modest volar oblique defects.
4. The treatment of the bone is instrumental. The distal volar bone is sculpted with small rongeurs. This step prevents hook-nail deformity and helps prevent dysesthesias.
5. Coverage of the denuded nail bed is not recommended, as this frequently leads to infection.

## INDICATIONS AND CONTRAINDICATIONS

The Atasoy flap is an especially useful flap well-known to hand surgeons^6^ and is made even more useful by the technical modifications to be described here. This triangular volar pad flap contains distal branches of the digital vessels and nerves that preserve sensibility,^2,7^ so it can be used to reconstruct fingertip amputations with exposed bone, as its subcutaneous tissue is carried with it. It can be used in severe pulp injuries, distal nail bed injuries, and amputations through the distal phalanx with loss of up to one-half of the phalanx, provided the loss is transverse or dorsal oblique (greater tissue loss dorsally). Although previously not recommended for use in volar oblique amputations, with these technique modifications, the Atasoy flap may be safely advanced further to resurface modest volar oblique losses. Care must always be taken to sufficiently mobilize the flap and advance it without tension.^7^

Fingertip amputations may be resurfaced with skin grafts if subcutaneous tissues have not been lost. Tissue loss may be treated with a cross-finger flap, provided sufficient sensibility of the contact pad remains.^3^ The V-to-Y advancement flap repair for fingertip injuries was first described by Atasoy et.al.^4^ in 1970. It demonstrated an excellent reconstructive method for single-stage repairs for many distal fingertip amputations with bone exposure except for cases with extensive soft-tissue loss or those with volar oblique amputations.

Conservative treatment consisting of dressing changes with possible skin grafting should be strongly considered in any patient with a high potential for poor wound healing. In patients with Raynaud’s disease, severe peripheral vascular disease, or with connective tissue diseases such as scleroderma, SLE, dermatomyositis, or CREST syndrome, this risk indicates that conservative treatment should be elected. Frequently, contracture of the wound as it heals by secondary intent makes the size of the skin graft quite small or unnecessary. In patients with diabetes mellitus with vascular involvement, consideration should be given to a surgical delay of the flap by incising through skin only.

## SURGICAL ANATOMY

The distal volar pad circulation and sensibility is provided by numerous branches of the paired digital arteries and nerves, which supply the volar pad from a point just distal to the distal interphalangeal (DIP) joint crease. The neurovascular branches extend fanlike from this point providing circulation and sensibility. Strong ligamentous attachments tether the volar pad skin to the underlying distal phalanx, especially along the radial and ulnar edges. Centrally, these fibrous septa are either absent or much weaker. Division of these lateral ligamentous fibers is imperative to obtain maximal, tension-free advancement of this extremely useful flap. The distal phalanx lies immediately volar to the nail plate and nail bed and must frequently be sculpted for ideal results.

## SURGICAL TECHNIQUE

The elevation of the flap was originally described as being through an incision through skin, creating a volar triangle, followed by dissection at the volar periosteum from a distal approach. Beasley^7^ noted the need for careful division of the vertical fibrous septa proximally for flap mobilization. It is imperative to divide these septa rather than to simply stretch them. One must be constantly mindful that the neurovascular elements enter the flap laterally and must be carefully preserved.

The technique of the V-Y flap described here differs from Atasoy’s original description. He indicated that dissection should begin at the distal traumatic edge of the flap and proceed proximally along the volar periosteum of the distal phalanx. It has been observed that this dissection does not produce any significant advancement. We recommend dissection at the mid flap level from lateral to medial.

Mobilization of the flap occurs when the ligamentous fibrous septa that tether the flap on either side are sharply divided. As these septa are intermingled with the terminal nerve branches and vascular elements, sharp dissecting scissors are indispensable.

In this description, treatment of the bone is underscored. By creating a gently sloping surface, a hypersensitive fingertip is avoided. Although dysesthesias due to stretching of the nerves has been reported, they are unlikely to occur with adequate mobilization. Proper bone treatment avoids hook-nail deformity and helps prevent dysesthesias.

Finally, this description of the flap recommends complete removal of the nail plate. This allows for visualization and repair of nail bed injuries. The nail plate should not be replaced, as it will create a nidus for infection. The new nail will grow normally without the need for a splint.

The steps which are useful in performing a safe, predictable Atasoy flap:

1) Severely damaged or necrotic skin, soft tissues, and nail bed should be meticulously debrided. The wound should be thoroughly irrigated. The denuded nail bed should be covered with a simple dressing that is removed at the first dressing change.

During debridement of the fingertip, obviously crushed or mangled soft tissues are meticulously excised with sharp dissecting scissors, preserving healthy, viable tissue. Crushed or detached bone is also debrided with small rongeurs. Even with the tourniquet in place, healthy tissue will demonstrate normal color and turgor. To evaluate tissue turgor, gentle pressure is exerted on the fibrofatty soft tissue. Healthy tissue will resist this pressure, whereas nonviable tissue will show little or no elasticity.

It is of utmost importance to carefully remove all necrotic and damaged tissue regardless of its location. Failure to do so presents an unacceptable risk of postoperative infection.

**Fig. 1.**
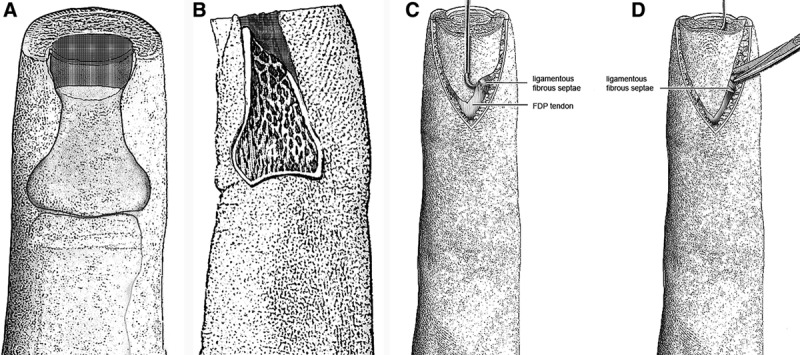
A, The shaded portion illustrates the volar portion of the distal phalanx to be excised. B, A lateral depiction is seen of the portion of the distal phalanx to be excised. C, The proximal dissection reveals the FDP tendon. There are no major neurovascular branches here. D, As the flap is placed under tension, the ligamentous fibrous septa are visualized. They can also be felt with the dissecting scissors.

**Fig. 2.**
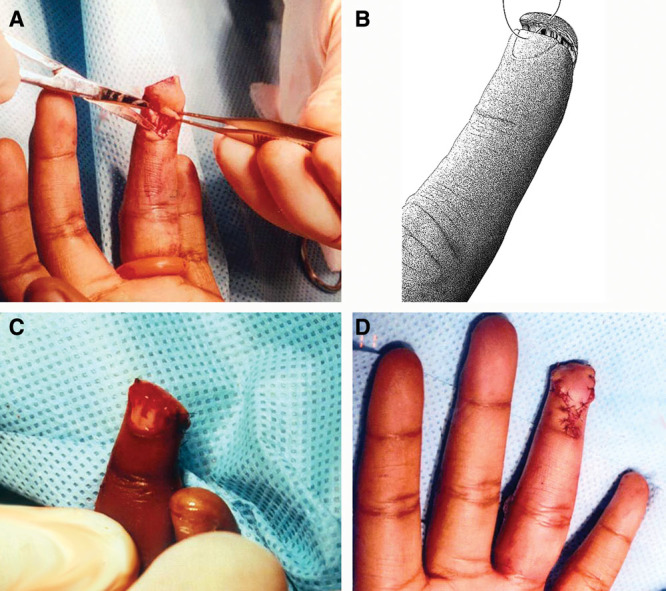
A, The edges of the flap are spread in a perpendicular direction, exposing the ligamentous fibrous septa by placing them under tension. B, The advancing edge is secured to the distal nail bed with 6-0 Vicryl interrupted sutures. C, The tourniquet has been released and satisfactory hemostasis has been obtained.

2) Bone treatment is essential. Small rongeurs are used to sculpt the distal volar bone to create a gently sloping surface from proximal to distal and if necessary, to remove more bone distally. The distal bone edge should be smooth. The distal nail bed should not extend beyond the distal edge of the distal phalanx (Fig. 1A, B).
3) Repair rents in the nail bed with 6-0 Vicryl sutures.
4) Flap planning is with a V incision with its apex at the center of the DIP joint crease. If one goes more proximal, the flap cannot be advanced. The ends of the V extend to the ulnar and radial corners of the nail bed. Incise skin full thickness and subcutaneous fat will protrude.
5) At the apex of the V, incise through subcutaneous tissue until the flexor digitorum profundus tendon sheath is exposed (Fig. 1C). This area has no major neurovascular branches and should be bluntly separated from the sides of the flap.
6) A small hook or fine Adson forceps under the distal flap edge should be used to place it under slight tension. Along the incised flap edges, the fibrous septa produce a check-reining action on the flap. These restraining ligaments can be felt with the dissecting scissors. The neurovascular elements appear more relaxed as they are submitted to less tension. The fibrous septa should be sharply divided with scissor tips only, sparing the neurovascular structures. A surprising degree of flap advancement results (Figs. 1D, 2A).
7) At the distal flap corners, cut through skin and soft tissue to bone. Sharply dissect the distal flap from underlying bone for 2–3 mm only.
8) Advance the flap without tension. Secure the advancing skin edge to the nail bed edge with 6-0 Vicryl simple interrupted sutures (Fig. 2B, C). Close the skin in Y fashion with 6-0 nylon simple interrupted sutures. Close the stem of the Y first, avoiding tension. It is preferable to leave gaps with protruding fat between sutures than to create suture line tension. Excise any excess skin corners to obtain a rounded fingertip.
9) The tourniquet should be released and the limb elevated. Apply mild compression until hemostasis is satisfactory. Apply a dressing. Splinting of the DIP joint in moderate extension may be applied at the surgeon’s discretion.
10) At the completion of the procedure, hemostasis is obtained by elevation and gentle compression for 3–5 minutes or until mild oozing is visible. If hemostasis cannot be achieved by this method, transection of a significant vessel should be suspected or, more likely, systemic hypertension. Adequate hemostasis may not be possible until the blood pressure is controlled. Efforts to control bleeding with cautery are contraindicated, as they serve to create areas of necrotic fat and/or skin with high risk of infection.

## POSTOPERATIVE CARE

It is common for the Atasoy flap to appear blanched immediately postoperatively. This subsides unless there is excessive tension or irreversible vascular damage. The nail plate is not replaced as this may lead to infection. Antibiotics active against *Staphylococcus aureus* should be prescribed for 7–10 days, with coverage for Gram-negative organisms recommended for food industry workers and landscapers, or when injuries occur in bodies of water. The prevalence of methicillin-resistant *S. aureus* should be considered, with a dressing change at 48 hours. Elevation of the extremity should always be stressed. Hydrogen peroxide is useful for release of an adherent surgical dressing. Nonabsorbable sutures should be removed after 10–14 days.

## PEARLS AND PITFALLS

A digital block using plain lidocaine 1% or 2% mixed 50:50 with plain bupivacaine 0.25% has been found to be efficacious. If circumstances permit, intravenous sedation is recommended. Note that bupivacaine is not recommended for use in children under 12 years old. Removing the nail plate and nail remnants removes contamination and allows the surgeon to see any previously unvisualized nail bed injuries. Curved iris “supercut” scissors are ideal for dissection. Sculpting of the volar phalanx with rongeurs helps prevent the dysesthesias occasionally seen with Atasoy flaps. The nail bed should extend to, but not beyond the bone. When closing skin, it is preferable to leave gaps with protruding fat between sutures than to create suture line tension. Dividing the V-Y flap transversely is not necessary and should be avoided, as this maneuver may endanger the original flap. The sensibility of these secondary flaps is routinely poor.^1^

It is essential to divide the fibrous septa after the skin incision is made. The technique presented here recommends closure of the donor defect as the stem of the “Y” allowing for primary wound healing. Dissection should not end when subcutaneous fat is visible, as this will not allow tension-free advancement. The solution is not to leave an open donor defect requiring dressing changes by the patient.^6^

The incision for the V-Y flap should extend to the DIP joint crease. This procedure should not be attempted by family physicians or other nonspecialists.^8^

Careful preservation of the distal neurovascular elements of the Atasoy flap is critical for maintaining the blood supply and sensibility of the flap. The digital arteries must never be ligated as described by Tranquilli-Leali.^2^

The flap technique described herein is superior to the V-Y ‘‘cup’’ flap because it creates equal advancement without the need for additional, potentially risky, dissection to the level of the PIP joint crease.^9^ Note that the distal corners of the V-Y flap cannot be rotated medially, as this will not only create a bilateral skin defects but also will create a dog-ear deformity centrally. The base (distal edge) of the flap does not need to be wider than the distal nail bed edge and in fact may be even 2–3 mm narrower with the technique described here.^10^

The flap described by Moberg,^11^ so useful for thumb injuries, is not advisable in the fingers because flexion of the IP joints is necessary for primary closure.^12^ This can lead to unacceptable permanent flexion deformities.

## COMPLICATIONS

Persistent tip tenderness is usually due to a prominent distal phalanx, which may require revision after soft-tissue healing is complete. Paresthesia or poor sensibility may be due to the traumatic injury or to iatrogenic injury to digital nerve branches. Frequently, however, it can be due to tension from inadequate flap mobilization. Flap necrosis is, fortunately, rare. It may be caused by iatrogenic injury to digital vessels or, more commonly, by unrecognized crush injury. During surgery, if unsure of circulation, one should release the tourniquet before insetting the flap; the tissue should bleed healthily. The nemesis, tension, must always be reduced or eliminated. When flap edge necrosis is seen, one should suspect tobacco smoke, directly or through second hand exposure. It may also be seen in the patient using nicotine replacement therapy or electronic cigarettes.

If the flap fails, all necrotic tissue must be sharply debrided regardless of its location. Nonviable bone must also be debrided. All exposed viable tissues should be treated with dressing changes until healing by secondary intent occurs, or until granulation tissue can be closed with a split thickness skin graft.

## CASE EXAMPLE

A 12-year-old right-handed Haitian girl was brought to my office by her father. She sustained a traumatic amputation of her left ring finger tip at home while using a sharp kitchen knife. X-rays demonstrated a transverse amputation of the distal one-third of the left ring finger distal phalanx. Medical history was unremarkable.

Examination revealed a transverse amputation of the left ring finger tip with slightly greater volar than dorsal loss. The remaining volar pad had good sensibility and circulation with 2-point discrimination of 5 mm radial and ulnar. The remaining nail plate was intact, with preservation of the proximal one-half of the nail bed.

After obtaining proper surgical consent, an Atasoy flap reconstruction was performed as described above. The distal aspect of the remaining volar phalanx was sculpted with small rongeurs to create a gently sloping surface. After dividing the ligamentous fibrous septa on either side of the flap, easy, tension-free advancement was obtained. Generous irrigation with normal saline was performed.

The distal flap edge was secured to the nail bed edge with interrupted simple sutures of 6-0 Vicryl. Skin was secured in Y fashion with interrupted simple sutures of 6-0 nylon.

Following removal of the tourniquet, hemostasis was gently obtained. Dressings and a splint were applied as described above. Duricef 500 mg daily was prescribed. On postoperative day 7, the dressings were changed. The flap demonstrated good color and sensibility. On postoperative day 14, the nylon sutures were removed and absorbable sutures along the distal nail bed left in place. The patient demonstrated full range of motion of her left ring finger and left hand. Two-point discrimination of the flap was 5 mm throughout.

## DISCUSSION

Although there are many well-designed studies demonstrating the efficacy of treatment of fingertip injuries with dressing changes,^13,14^ the benefits of primary closure should not be overlooked. Some of these advantages are decreased pain, more rapid healing (10 days versus 30 days), better preservation of length, lower risk of dysesthesias, lower risk of cold intolerance, avoidance of hook-nail deformity, and immediate coverage of exposed bone. In the case of a fingertip amputation involving the distal phalanx with transverse, dorsal oblique, or even modest volar oblique orientation, the technique presented here is ideally suited for primary closure.

With the technique modifications and recommendations presented here, the Atasoy flap can be performed with predictably excellent results.

1) The original description is for the direction of dissection to be from distal to proximal. Here, the direction of dissection is shifted to be from lateral to medial.
2) The plane of dissection is shifted from the flexor tendon sheath and periosteum to the mid flap plane.
3) Careful lysis of the fibrous ligamentous septa is emphasized. This dissection allows the Atasoy flap to be advanced further.
4) The treatment of the bone is emphasized to prevent dysesthesias and hook-nail deformity.
5) Coverage of the nail bed is not recommended.

This flap is an anatomical, single-stage reconstruction with virtually no donor-site morbidity. It can be a workhorse for the treatment of a variety of fingertip injuries, including amputations through the distal phalanx. The disadvantages of this flap are few. The flap may fail. Dysesthesias may occur.

The advantages and disadvantages of primary closure versus healing by secondary intent should be presented to patients when obtaining informed consent. Regardless of one’s preference for the treatment of fingertip injuries, the Atasoy flap should be a part of every surgeon’s armamentarium.

## ACKNOWLEDGMENT

Dr. Viciana wishes to gratefully acknowledge the assistance of Robert W. Beasley, MD, in the preparation of the article.
